# Rolipram Improves Outcome in a Rat Model of Infant Sepsis-Induced Cardiorenal Syndrome

**DOI:** 10.3389/fphar.2017.00237

**Published:** 2017-05-03

**Authors:** Clark R. Sims, Sharda P. Singh, Shengyu Mu, Neriman Gokden, Dala Zakaria, Trung C. Nguyen, Philip R. Mayeux

**Affiliations:** ^1^Department of Pharmacology and Toxicology, University of Arkansas for Medical Sciences, Little RockAR, USA; ^2^Department of Pathology, University of Arkansas for Medical Sciences, Little RockAR, USA; ^3^Department of Pediatrics, Division of Pediatric Cardiology, University of Arkansas for Medical Sciences and Arkansas Children’s Hospital, Little RockAR, USA; ^4^Section of Critical Care Medicine, Department of Pediatrics, Baylor College of Medicine and Center for Translational Research on Inflammatory Diseases, Michael E. DeBakey Veterans Affairs Medical Center, HoustonTX, USA

**Keywords:** sepsis, cardiorenal syndrome, infant, rat, rolipram

## Abstract

While the mortality rate associated with sepsis in children has fallen over the years, it still remains unacceptably high. The development of both acute cardiac dysfunction and acute kidney injury during severe sepsis is categorized as type 5 cardiorenal syndrome (CRS) and is poorly understood in infants. To address this lack of understanding and the need for an appropriate animal model in which to conduct relevant preclinical studies, we developed a model of infant sepsis-induced CRS in rat pups then evaluated the therapeutic potential of the phosphodiesterase (PDE) 4 inhibitor, rolipram. Rat pups at 17–18-days old were subjected to cecal ligation and puncture (CLP) to induce fecal polymicrobial sepsis. Uptake of Evans Blue dye was used to assess renal microvascular leakage. Intravital videomicroscopy was used to assess renal microvascular perfusion and oxidant generation. Glomerular filtration rate (GFR) was used to assess renal function. Left ventricular (LV) catheterization and echocardiography were used to assess cardiac function. Impairment of both cardiac and renal function developed rapidly following CLP, indicating type 5 CRS. Most notable were the rapid decline in LV diastolic function, the decline in cardiac output, renal microvascular failure, and the decline in GFR. A dose-response study with rolipram determined 0.1 mg/kg, ip as the lowest most efficacious dose to protect the renal microcirculation. Rolipram was then evaluated using a clinically relevant delayed dosing paradigm (a single dose at 6 h post-CLP). With delayed dosing, rolipram restored the renal microcirculation and reduced microvascular leakage but did not reduce oxidant generation in the kidney nor restore GFR. In contrast, delayed dosing with rolipram restored cardiac function. Rolipram also improved 4-days survival. In summary, CLP in the rat pup produces a clinically relevant pediatric model of sepsis-induced CRS. The PDE4 inhibitor rolipram was effective in improving renal microvascular function and cardiac function, which improved mortality. These findings suggest that rolipram should be evaluated further as adjunctive therapy for the septic infant with CRS.

## Introduction

In the United States, sepsis is the 6th and 7th leading cause of death in neonates and infants, respectively (Heron, 2015). While the mortality rate associated with sepsis in children has fallen over the years, it still remains unacceptably high at 5–25% (Hartman et al., 2013; Ruth et al., 2014; Weiss et al., 2015). Severe sepsis is defined as sepsis with cardiovascular distress and multiple organ dysfunction syndrome (MODS), which increases morbidity and mortality in this understudied patient population (Goldstein et al., 2005; Weiss et al., 2015).

The heart and kidneys are two organs commonly affected by sepsis. The development of both acute cardiac dysfunction and acute kidney injury during severe sepsis is categorized as type 5 CRS (McCullough et al., 2013). The etiology of sepsis-induced CRS in infants is poorly understood (Jefferies and Goldstein, 2013) but appears to be different than that in adults (Aneja and Carcillo, 2011; Deep et al., 2013; Ranjit et al., 2014; Zaky et al., 2014). For example, adults with septic shock typically present in a hyperdynamic state with high cardiac output and low systemic vascular resistance (warm shock) whereas infants more often exhibit low cardiac output and high systemic vascular resistance (cold shock) (Aneja and Carcillo, 2011). Treatment for sepsis-induced CRS focuses on interventions for the underlying systemic cause while considering functional support for the accompanying cardiac and renal dysfunctions (Brophy, 2008; de Oliveira et al., 2008; de Oliveira, 2010). Nevertheless, mortality rates for these critically ill children with CRS can still approach 50% (Romanovsky et al., 2013). The reason for poor outcomes is related to the fact that treatments are not specifically targeted and what supportive care is available is usually begun only after symptoms are present and after organ injury may have already been initiated (Simmons et al., 2012; Fortenberry et al., 2013).

Phosphodiesterase catalyzes the hydrolysis of cAMP and cGMP. In the cardiovascular system, increased levels of cAMP or cGMP can decrease vascular tone, strengthen endothelial tight junctions, and increase cardiac contractility. Tissue distribution of the large PDE superfamily is generally broad; however, differences in their regulation and selectivity (or not) for cAMP and cGMP can be exploited pharmacologically using selective PDE inhibitors (Maurice et al., 2014). For example, in the volume-resuscitated septic infant with acute cardiac dysfunction, PDE3 inhibitors increase levels of both cAMP and cGMP to improve cardiac contractility (Barton et al., 1996; Irazuzta et al., 2007; Meyer et al., 2011) and increase survival (de Oliveira et al., 2008; Brierley et al., 2009). PDE4 is selective for cAMP (Boswell-Smith et al., 2006; Maurice et al., 2014) and in adult animal model of sepsis, inhibitors of PDE4 reduce systemic vascular resistance (Carcillo et al., 1996), improve cardiac contractility (Thomas et al., 2003) and improve renal function (Carcillo et al., 1996; Tanahashi et al., 1999; Holthoff et al., 2013). PDE4 inhibitors also have potent anti-inflammatory activity and reduce microvascular leakage (Sanz et al., 2007; Schick et al., 2012), all of which could have added benefits in infants with severe sepsis.

The pathophysiology of sepsis-induced-CRS in infants is poorly understood due to limited studies in this patient population and the paucity of studies that have examined sepsis-induced MODS in age-appropriate animal models. To address this lack of understanding and the need for an appropriate animal model in which to conduct relevant preclinical studies, we developed a model of infant sepsis-induced cold shock in rat pups subjected to CLP (Seely et al., 2011). Here, we describe the development of type 5 CRS in this model of infant sepsis and then use of this model to evaluate the therapeutic potential of the PDE 4 inhibitor, rolipram.

## Materials and Methods

### Animals

All animals were housed and handled in accordance to the National Institutes of Health Guide for the Care of Laboratory Animals with approval from the University of Arkansas for Medical Sciences Institutional Animal Care and Use Committee and the Central Arkansas Veterans Healthcare System Institutional Animal Care and Use Committee. Seven 10-day-old Sprague Dawley rat pups along with a dam (Charles River Laboratories, Wilmington, MA, USA) were allowed to acclimatize for 7 days with free access to the dam. All experiments were performed when the rat pups were between 17 and 18 days old (mean weight of 49.4 ± 0.4 g; *n* = 221). With weaning as the scaling factor, rat pups between 17 and 18 days old can be roughly estimated to be at a stage of development comparable to a 5- to 6-month-old human infant (Quinn, 2005; Andreollo et al., 2012).

### Animal Model of Sepsis

Cecal ligation and puncture to induce severe sepsis was performed as described previously (Seely et al., 2011; Sims et al., 2014). While the pups were under isoflurane anesthesia, the cecum was exposed through a midline incision and 1.5 cm of the cecal tip was ligated using 4-0 silk suture. The cecum was punctured through and through with an 18-gauge needle and approximately 1 mm of fecal material was expressed through each opening. In non-septic control pups (Sham), a midline incision was made but the cecum was neither ligated nor punctured. At the time of surgery, all pups were given 25 ml/kg of prewarmed saline to replace lost fluids and buprenorphine (0.01 mg/kg, sc) and thereafter as needed to reduce pain. The rat pups were then placed in cages on a heating pad with access to DietGel^®^ Recovery nutrient gel (ClearH2O, Westbrook, ME, USA) as well a pellet chow and water. Rat pups studied at time points longer than 6 h received imipenem/cilastatin (14 mg/kg) in normal saline (40 ml/kg, sc) at 6 h. Temperature was recorded using a rectal thermometer.

### Assessment of the Renal Microcirculation and Mitochondrial Oxidant Generation

Following anesthesia with isoflurane, a tail vein injection containing fluorescein isothiocyanate-labeled dextran (FITC-Dextran, 2 μmol/kg in 3 ml/kg normal saline) and MitoSOX Red Superoxide Indicator (Molecular Probes, Eugene, OR, USA) was administered to visualize the capillary space and detect mitochondrial superoxide generation, respectively, using intravital videomicroscopy as described previously (Seely et al., 2011; Holthoff et al., 2013; Sims et al., 2014). After 10 min, the left kidney was exposed by a flank incision and the animal was positioned on a glass stage above an inverted Zeiss Axiovert 200M fluorescent microscope equipped with an Axiocam HSm camera (Zeiss, Germany). Videos of 10 s (approximately 30 frames/second) and images (500 ms exposure) at 200× magnification were acquired from five non-overlapping fields of view. From these videos, approximately 150 capillaries were analyzed from the kidney of each rat pup. Randomly selected capillaries from each of the videos were categorized as having continuous flow, where RBC movement was uninterrupted; intermittent flow, where RBC movement stopped or reversed; or no flow, where no RBC movement was observed. Data are expressed as the percentage of vessels in each of the three categories. RBC velocity through the microcirculation was calculated using only capillaries with continuous flow by measuring the distance a single RBC traveled over time. MitoSOX fluorescence in renal tubules was calculated from the still images using ImageJ (National Institutes of Health, Bethesda, MD, USA) and is expressed in arbitrary units per μm^2^.

### Lactate and Blood Urea Nitrogen (BUN)

Lactate and BUN concentrations were measured using the Lactate (Liquid) Reagent Set (Pointe Scientific Inc., Canton, MI, USA) and the QuantiChrom Urea Assay kit (BioAssay Systems, Hayward, CA, USA) respectively. Data are expressed as mmol/L for lactate and BUN.

### Glomerular Filtration Rate (GFR)

Glomerular filtration rate was determined using a small transcutaneous fluorimeter (NIC-Kidney Device, Mannheim Pharma & Diagnostic, Germany) (Schreiber et al., 2012; Ellery et al., 2015). Under isoflurane anesthesia, a small region on the back of the rat pup was shaved and the small device was attached using surgical grade double-sided adhesive tape and secured using surgical self-adhesive tape. After a brief baseline recording, fluorescein isothiocyanate-labeled sinistrin (FITC-Sinistrin, 7 mg/100 mg body weight) was administered via the tail vein and the animals were allowed to awaken. Transcutaneous fluorescence was recorded every 2 s over a 2-h period and GFR was calculated from the FITC-Sinistrin clearance rate. Data are presented as ml/min/100 g of bodyweight.

### Left Ventricular (LV) Function

Rats (sham or after CLP as indicated above) were anesthetized using isoflurane with spontaneous breathing and maintained heart rate at 350–400 bpm during the procedure. A 1.2F pressure catheter (Transonic, Ithaca, NY, USA) was introduced from the right carotid artery (retrograde) through the aortic valve to reach the cavity of the LV. LV pressure was recorded for 15–30 min using the SP-200 system (Transonic) and analyzed using LabChart Pro8 software. The rate of LV pressure rise during systole (dP/dt max), the maximal negative slope at the isovolumic relaxation phase (dP/dt min), and LV relaxation time constant (Tau) were derived from the pressure waves.

### Echocardiography

Echocardiography was performed 6 or 18 h after CLP or sham surgery using a Vevo 770 imaging system and RMV707B transducer (30 MHz) (VisualSonics, Toronto, ON, Canada). The rat pups were placed on a heating platform to maintain body temperature at 37 ± 0.5°C. Anesthesia was maintained with isoflurane at 1.5–2.0% mixed with O_2_ and continuously adjusted so that heart rate remained at 350–410 bpm throughout the procedure. Short axis M-mode recordings at the mid left LV level were used to obtain LV wall thickness, inner diameter and volume, and the functional parameters ejection fraction, fractional shortening, stroke volume, and cardiac output.

### Microvascular Leakage

Renal microvascular leakage was assessed using EBD (Sigma–Aldrich) as previously described (Seely et al., 2011; Holthoff et al., 2013). Briefly, at 18 h after CLP, the rat pups were anesthetized with isoflurane and injected with EBD (1% solution, wt/vol, in saline at 2 ml/kg) via the tail vein and allowed to wake up. After 30 min, the animals were anesthetized again and perfused with PBS through the LV until all blood was eliminated. The right kidney was rapidly removed, weighed, and stored at -80°C until homogenization in 1 ml formamide and incubation for 18 h at 55°C. The amount of EBD in the supernatant was analyzed by measuring absorbance at 620 nm against a standard curve. Data are expressed as micrograms of EBD per milligram of kidney tissue (μg EBD/g tissue).

### Morphology

The LV and left kidney of each animal were fixed in 10% phosphate-buffered formalin and embedded in paraffin. Sections (5 μm) of heart were cut and stained with H&E or PAS for section of kidney for analysis of morphology. Morphological analysis was performed with light microscopy (Nikon E800, Nikon, Melville, NY, USA) by a blinded observer.

### Cardiac Troponin I (cTnI)

Cardiac troponin I was assessed in plasma drawn from the vena cava using a rat cTnI ELISA (Life Diagnostics, West Chester, PA, USA). Data are expressed in ng/ml of plasma.

### Survival Studies

Core body temperature was used as an indicator of pending death (Warn et al., 2003; Patil et al., 2014). It was measured using a rectal temperature probe every 4 h beginning 6 h post-CLP surgery. At 6 h post-CLP surgery, the first dose of antibiotics (imipenem/cilastatin, 14 mg/kg in saline) and fluids (saline at 40 ml/kg) were administered. After the first 24 h, the heating pad was removed and temperature was measured every 8 h up to 96 h. At 18 and 30 h post-CLP a lower dose of imipenem/cilastatin (7 mg/kg in 40 ml/kg saline) was administered. Pups were considered non-survivors if they died or had to be euthanized when two consecutive readings of core body temperature were <28.0°C.

### Rolipram

A stock solution of rolipram (Cayman Chemical, Ann Arbor, MI, USA) was prepared by dissolving 10 mg in 1 ml of DMSO. Just prior to administration, the stock solution was diluted in normal saline such that the desired dose was administered by ip injection in a volume of 2 μl/g body weight containing 1% DMSO (vehicle). For example, the typical pup weighed 50 g (mean pup weight was 49.5 ± 0.4 g; *n* = 221) and would have received 100 μl. We previously showed that 1% DMSO vehicle does not affect CLP-induced changes in renal microvascular perfusion, leakage or GFR in the mouse (Holthoff et al., 2013). We confirmed that vehicle did not affect renal perfusion in rat pups subjected to CLP. Because the potential effects of DMSO vehicle on cardiac function have never been evaluated in the rat pup, vehicle groups were included in the experimental design.

### Statistical Analysis

All data are presented as mean ± standard error of mean (SEM) and were analyzed using Prism 6 (GraphPad Software, San Diego, CA, USA). The Student’s *t*-test was used when two groups were compared and a one-way analysis of variance (ANOVA) followed by the Tukey *post hoc* test was used when three or more groups were compared. A two-way ANOVA followed by the Dunnett *post hoc* test was used to evaluate the dose-response study. Survival curves were analyzed using the Log-rank test. A *p-*value < 0.05 was considered a significant difference between groups.

## Results

### Time Course Studies

**FIGURE 1:**
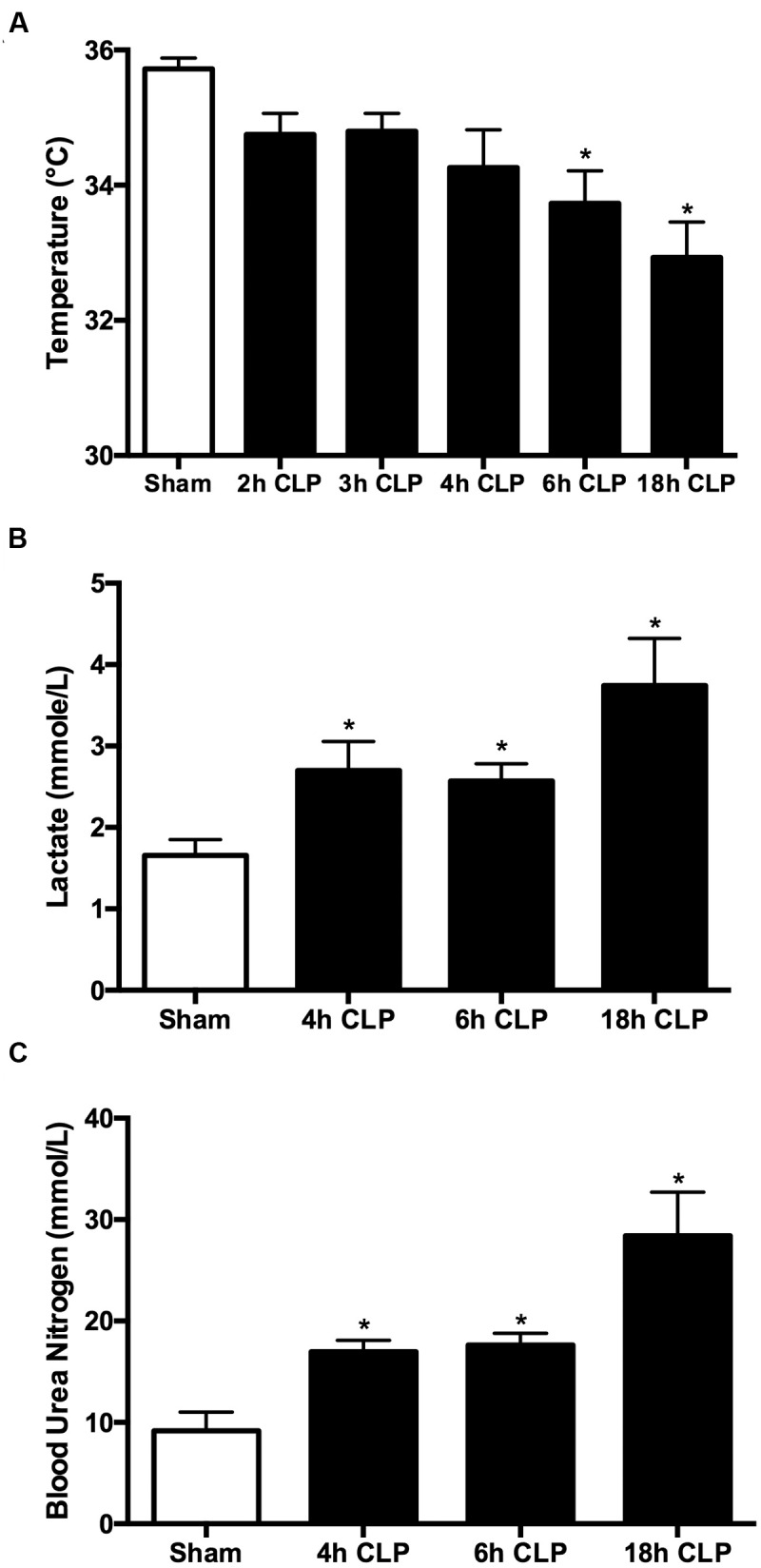
**Time course for clinical markers of sepsis.** All animals received antibiotics and fluids at 6 h post-CLP (see Materials and Methods). The decline in core temperature reached significance at 6 h **(A)**; ^∗^*p* < 0.05 compared to Sham (*n* = 25, a mixture of sham at all time points; *n* = 6–23 for CLP animals). Plasma lactate was significantly elevated by 4 h **(B)**; ^∗^*p* < 0.05 compared to Sham (*n* = 14, a mixture of Sham at all time points; *n* = 6–13 for CLP animals). BUN was significantly elevated by 4 h **(C)**; ^∗^*p* < 0.05 compared to Sham (*n* = 9, a mixture of Sham at all time points; *n* = 7–8 for CLP animals).

In rodents, temperature can be used as an indicator of the systemic inflammatory response syndrome (Warn et al., 2003). Core temperature decreased in pups subjected to CLP as sepsis developed, reaching significance compared to Sham pups at 6 and 18 h (**Figure 1A**). Levels of lactate in the CLP group increased over time compared to Sham group reaching significance by 4 h post-CLP and a 2.3-fold increase over the Sham group at 18 h (**Figure 1B**). These findings are consistent with a decline in microvascular perfusion and possible cardiovascular dysfunction (Goldstein et al., 2005; Casserly et al., 2015). BUN was also elevated in a similar time course (**Figure 1C**), which suggests a possible decline in renal function (Brophy, 2008).

**FIGURE 2:**
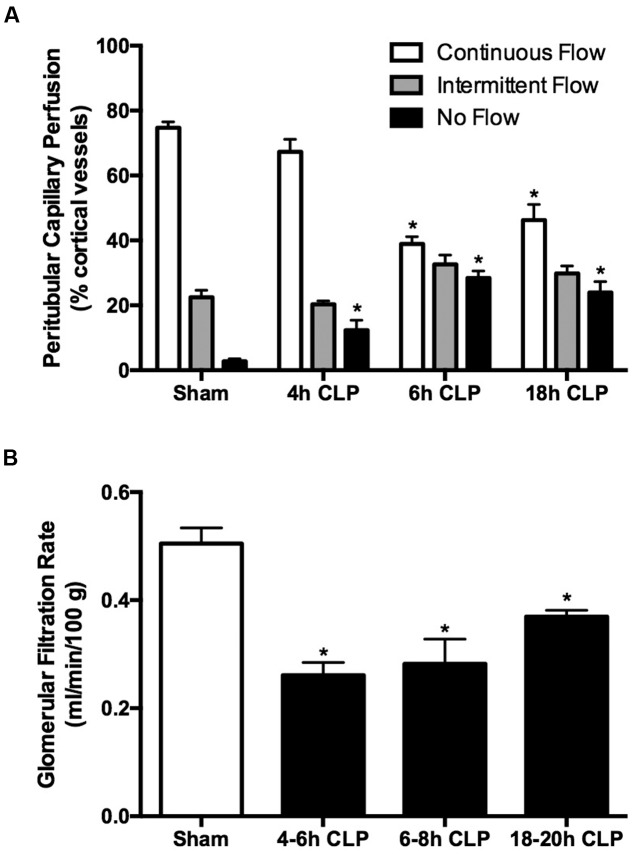
**Time course of sepsis-induced changes in renal microvascular perfusion and GFR.** Cortical peritubular perfusion was dramatically reduced by 6 h and remained decreased through 18 h **(A)**; ^∗^*p* < 0.05 compared to Sham (*n* = 13, a mixture of Sham at all time points; *n* = 5–7 for CLP animals). Glomerular filtration rate was significantly decreased at 4 h and remained decreased through 18 h **(B)**; ^∗^*p* < 0.05 compared to Sham (*n* = 9, a mixture of Sham at all time points; *n* = 5–7 for CLP animals).

Renal cortical microvascular perfusion was assessed using intravital videomicroscopy with FITC-dextran to visualize RBC movement in cortical peritubular capillaries. At the earliest time point measured, 4 h, the percentage of capillaries with no flow was significantly increased in pups subjected to CLP compared to the Sham group (**Figure 2A**). By 6 h, the percentage of capillaries with continuous flow was decreased by nearly 50% and the overall decline in perfusion was sustained through 18 h. The decrease in GFR, estimated by FITC-sinistrin clearance, paralleled the decline in cortical microvascular perfusion. At 6 h GFR was decreased by 50% in pups subject to CLP and remained decreased through 18 h (**Figure 2B**). These data indicated the very rapid development of renal microvascular dysfunction and acute renal dysfunction in septic pups.

### Assessment of Cardiac Function

**FIGURE 3:**
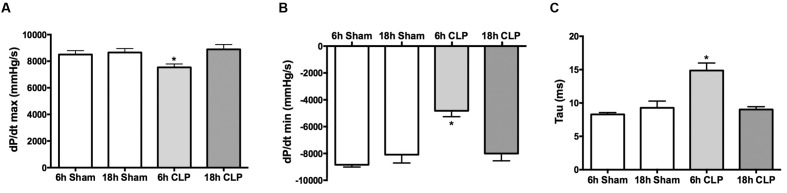
**Time course for changes in LV function.** At 6 h but not at 18 h post-CLP, dP/dt max **(A)**, dP/dt min **(B)**, and Tau **(C)** were significantly different from the time-matched Sham group; ^∗^*p* < 0.05; *n* = 3 for 18 h Sham; *n* = 5 for all other groups.

Left ventricular function was assessed in pups by analyzing LV pressure waves using a pressure catheter advanced into the LV from the right carotid artery. The rate of LV pressure rise during systole (dP/dt max) was slightly but significantly decreased (10%) at 6 h post-CLP compared to the Sham group (**Figure 3A**). The maximal negative rate of pressure fall during the diastolic isovolumic relaxation phase (dP/dt min) was significantly decreased by 45% (**Figure 3B**) and the LV relaxation time constant (Tau) was significantly increased by 77% (**Figure 3C**) at 6 h compared to the Sham group. At 18 h (in separate groups of pups), these measures of LV function were not different from the Sham group. Hematocrits were not different between Sham and CLP groups at 6 h (32 ± 2 and 33 ± 2%, respectively) and 18 h (24 ± 2 and 25 ± 1%, respectively). At 6 h post-CLP, plasma cTnI levels were increased in the CLP group (0.56 ± 0.13 ng/ml, *n* = 7) compared to the Sham group (0.22 ± 0.04 ng/ml, *n* = 6, *p* < 0.05).

**FIGURE 4:**
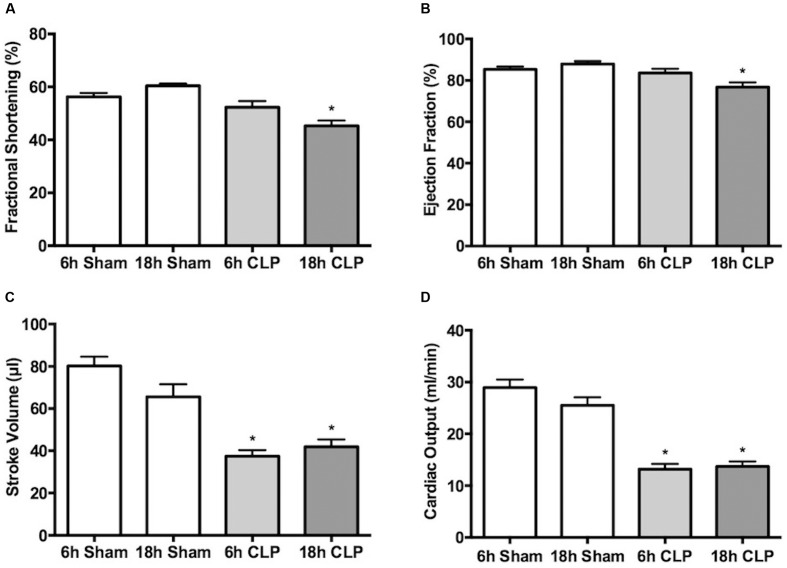
**Time course of changes in echocardiography.** Fractional shortening **(A)**, ejection fraction **(B)**, stroke volume **(C)** and cardiac output **(D)** were measured in separate groups of animals at 6 or 18 h. At 6 h post-CLP, stroke volume and cardiac output were significantly decreased compared to Sham; ^∗^*p* < 0.05; *n* = 5 for 6 h Sham; *n* = 9 for 6 h CLP. AT 18 h post-CLP, all parameters were significantly decreased compared to the time-matched Sham groups; ^∗^*p* < 0.05; *n* = 9 for 18 h Sham; *n* = 10 for 18 h CLP.

In separate groups of pups, echocardiography was used to assess cardiac function non-invasively at 6 and 18 h post-CLP or sham surgery. Fractional shortening (**Figure 4A**) and ejection fraction (**Figure 4B**) were unaffected at 6 h but were significantly decreased at 18 h. In contrast, stroke volume (**Figure 4C**) and cardiac output (**Figure 4D**) were decreased nearly 50% at 6 and 18 h post-CLP (**Figure 4D**).

### Histology of Kidney and Heart

**FIGURE 5:**
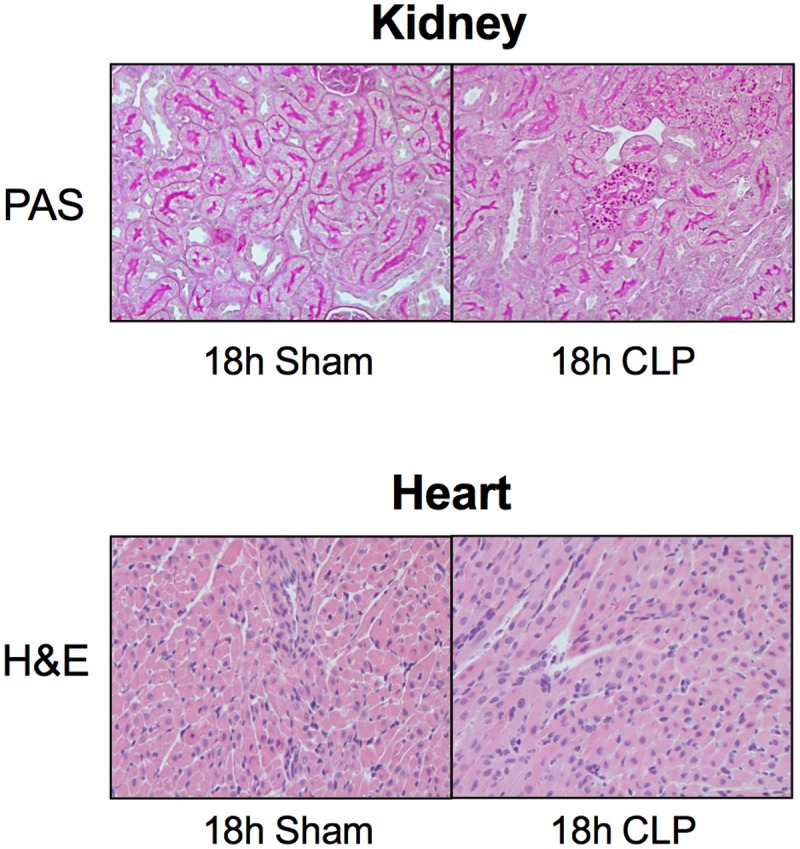
**Histology of the kidney and heart.** Shown are representative images of the kidney stained with PAS staining and the heart stained with H&E. No morphological changes were observed between Sham and CLP groups (*n* = 4 per group) other than patchy PAS-positive cytoplasmic droplets within proximal tubules of the kidney. Original magnification = 200×.

Short-term models of sepsis in rodents typically produce very mild histological changes (Seely et al., 2011; Smeding et al., 2012; Holthoff et al., 2013). Neither the kidney nor LV showed evidence of necrosis. Proximal tubules in the kidney cortex of the CLP group at 18 h did show evidence of patchy PAS-stained cytoplasmic droplets within the tubular epithelium (**Figure 5**).

### Rolipram Dose-Response Study

**FIGURE 6:**
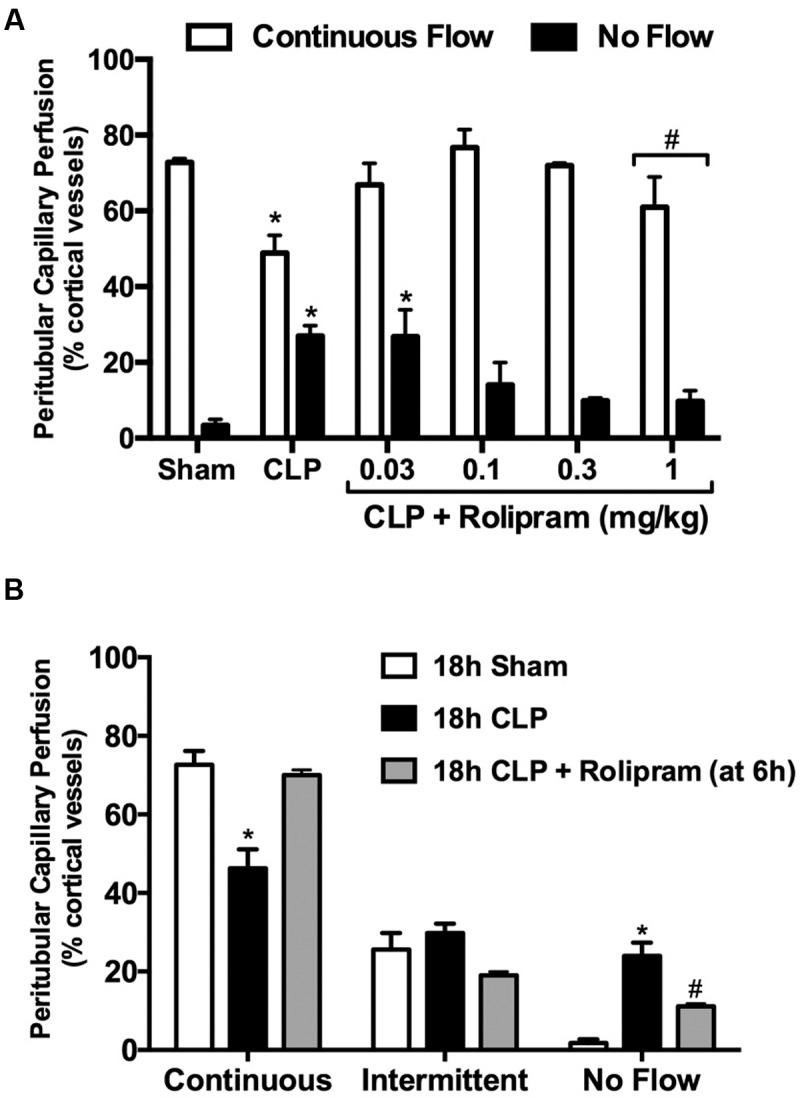
**Effects of rolipram on renal microvascular perfusion.** A dose-finding study **(A)** was used to determine the efficacy of rolipram to prevent the decline in renal cortical microvascular perfusion. Rolipram was administered at the time of CLP (ip injection) and perfusion was measured at 6 h; *n* = 5 for Sham; *n* = 8 for CLP; *n* = 5–8 for CLP + Rolipram; ^∗^*p* < 0.05 compared to Sham by two-way ANOVA; ^#^this dose produced 70% lethality. The dose of 0.1 mg/kg was the lowest dose to prevent both the decline in vessels with continuous flow and the increase in vessels with no flow. Next, rolipram (0.1 mg/kg, ip) was administered at 6 h post-CLP and perfusion was measured at 18 h **(B)**. Even with delayed administration, rolipram protected renal microvascular perfusion; ^∗^*p* < 0.05 compared to Sham and CLP + Rolipram; ^#^*p* < 0.05 compared to Sham and CLP; *n* = 5 for 18 h Sham; *n* = 6 for 18 h CLP; *n* = 5 for 18 h CLP + Rolipram.

As a PDE4 inhibitor, rolipram can reduce vascular resistance (Carcillo et al., 1996) and improve microvascular perfusion (Holthoff et al., 2013). We reported previously that in adult aged mice subjected to CLP, the lowest most efficacious dose of rolipram that preserves renal peritubular capillary perfusion is 1 mg/kg, ip (Holthoff et al., 2013). When this dose was administered to pups at the time of CLP, 5 of 7 pups died (70% lethality) by 6 h. Doses of 0.03, 0.1, and 0.3 mg/kg (ip) were then tested (**Figure 6A**). The dose of 0.1 mg/kg was the lowest dose that prevented both the decline in capillaries with continuous flow and the increase in capillaries with no flow, with no apparent toxicity. The dose of 0.1 mg/kg was then used in subsequent experiments. In addition to the decline in renal microvascular perfusion, serum levels of the inflammatory cytokines TNF-α and IL-1β are already elevated by 6 h (Seely et al., 2011). Also, core temperature is decreased (**Figure 1A**) and plasma lactate levels are elevated (**Figure 1B**) at 6 h post-CLP, the time when antibiotics and fluids (40 ml/kg saline, sc) are administered. Since therapy for the septic infant is begun only after the onset of symptoms, a delayed dosing paradigm with rolipram was tested to more closely mimic the clinical setting. Rolipram (0.1 mg/kg, ip) was administered 6 h following CLP and peritubular capillary perfusion was evaluated at 18 h. Even this delay in therapy restored peritubular capillary perfusion at 18 h (**Figure 6B**).

### Effects of Rolipram on Renal and Cardiac Function

**FIGURE 7:**
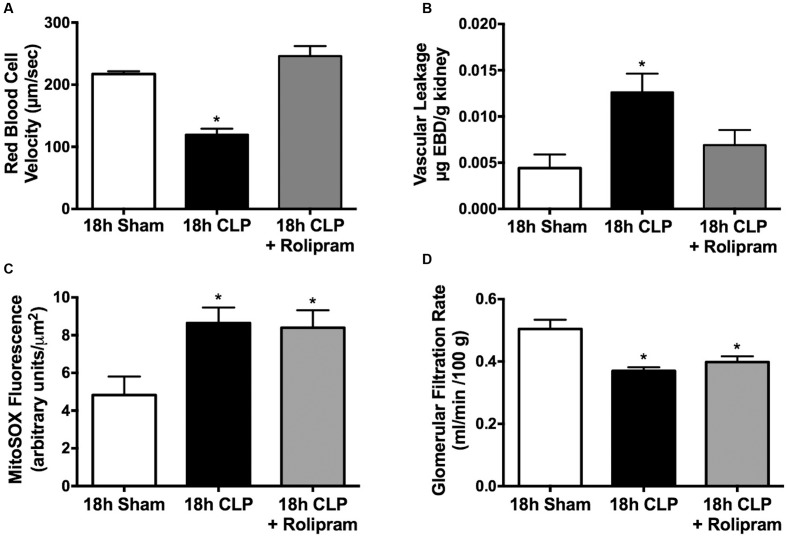
**Effects of delayed rolipram dosing on the renal cortical peritubular microenvironment and renal function.** Rolipram (0.1 mg/kg, ip) was administered at 6 h post-CLP. Rolipram protected against the CLP-induced decline in RBC velocity in capillaries with continuous flow **(A)**; ^∗^*p* < 0.05 compared 18 h Sham and 18 h CLP + Rolipram; *n* = 5 in all groups. Rolipram also protected against the CLP-induced increase in microvascular leakage **(B)**; ^∗^*p* < 0.05 compared 18 h Sham and 18 h CLP + Rolipram; *n* = 4–6 per group. However, rolipram had no effect on the CLP-induced renal tubule MitoSOX fluorescence, an indicator of mitochondrial oxidant generation **(C)**; ^∗^*p* < 0.05 compared to 18 h Sham; *n* = 4–5 in all groups. Rolipram also had no effect on the CLP-induced decline glomerular filtration **(D)**; ^∗^*p* < 0.05 compared to 18 h Sham; *n* = 4–9 in all groups.

In addition to the decline in the percentage of cortical peritubular capillaries with continuous flow at 18 h post-CLP, there was a 50% decline in RBC velocity in capillaries with continuous flow and rolipram, administered at 6 h post-CLP, prevented this decline (**Figure 7A**). Microvascular leakage and oxidative stress are hallmarks of sepsis, which can have a negative impact on organ function. We showed previously that rat pups subjected to CLP develop renal microvascular leakage (Seely et al., 2011). PDE4 inhibitors can reduce inflammatory microvascular leakage (Schick et al., 2012) and rolipram administered at 6 h post-CLP did prevent CLP-induced renal capillary leakage at 18 h (**Figure 7B**). We also showed previously that rat pups subjected to CLP develop mitochondrial oxidant generation (Sims et al., 2014) and oxidative stress (Seely et al., 2011) in renal tubules. Rolipram, administered at 6 h post-CLP did not affect MitoSOX fluorescence, an indicator of mitochondrial oxidant generation (**Figure 7C**). Both reduced microvascular perfusion and oxidative stress can have a negative impact on renal function (Wang et al., 2012; El-Achkar and Dagher, 2015; Matejovic et al., 2015); therefore, we tested the effects of rolipram on GFR. Despite preventing renal cortical microvascular dysfunction, rolipram did not improve GFR (**Figure 7D**).

**FIGURE 8:**
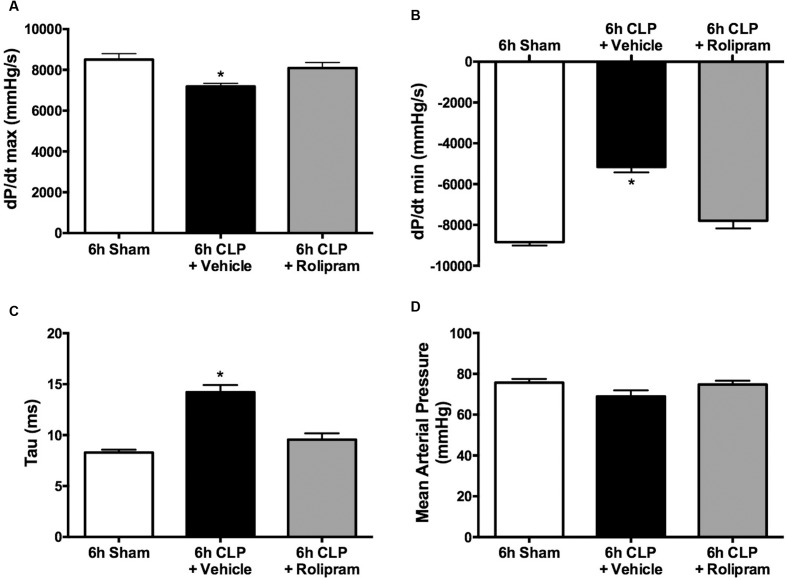
**Effects of rolipram on LV function and blood pressure.** Rolipram (0.1 mg/kg, ip) was administered at the time of CLP and LV function and blood pressure were measured at 6 h. Rolipram protected against the CLP-induced decline in dP/dt max **(A)**, the decline in dP/dt min **(B)**, and the increase in Tau **(C)** without affecting mean arterial pressure **(D)**; ^∗^*p* < 0.05 compared to 6 h Sham and 6 h CLP + Rolipram; *n* = 5–6 in all groups.

Since we observed a decline in LV function at 6 h but not at 18 h post-CLP, the effects of rolipram (0.1 mg/kg, ip) were evaluated at 6 h. For these experiments, rolipram was administered at the time of CLP. Rolipram prevented the decrease in dP/dt max, the decrease in dP/dt min, and the increase in Tau (**Figures 8A–C**). Because blood pressure can affect these measures of LV function, mean arterial pressure was measured prior to advancing the catheter to the LV. There was no difference in mean arterial pressure in the CLP group compared to Sham (**Figure 8D**), as we reported previously in awake pups (Seely et al., 2011). Rolipram had no effect on mean arterial pressure, suggesting this dose of rolipram was low enough not to compromise control of systemic vascular resistance, which could have worsened organ perfusion. Together these data indicate that rolipram protected against the early decline in LV function.

**FIGURE 9:**
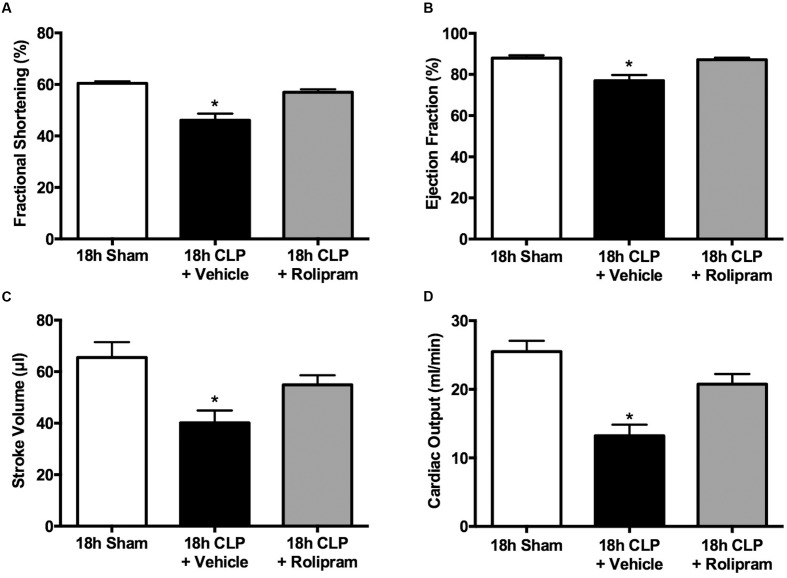
**Effects of delayed rolipram dosing on echocardiography.** Rolipram (0.1 mg/kg, ip) was administered at 6 h post-CLP. Rolipram protected against the CLP-induced decline fractional shortening **(A)**, ejection fraction **(B)**, stroke volume **(C)** and cardiac output **(D)**; ^∗^*p* < 0.05 compared 18 h Sham and 18 h CLP + Rolipram; *n* = 7–14 in all groups.

Because we observed a decline in all echocardiography parameters at 18 h post-CLP, we used the delayed dosing paradigm to evaluate the therapeutic potential of rolipram using echocardiography. When administered at 6 h post-CLP, a time when stroke volume was already decreased (**Figure 4C**), rolipram (0.1 mg/kg, ip) prevented the decline in fractional shortening and ejection fraction, and restored stroke volume and cardiac output (**Figures 9A–D**).

### Effects of Rolipram on Survival

**FIGURE 10:**
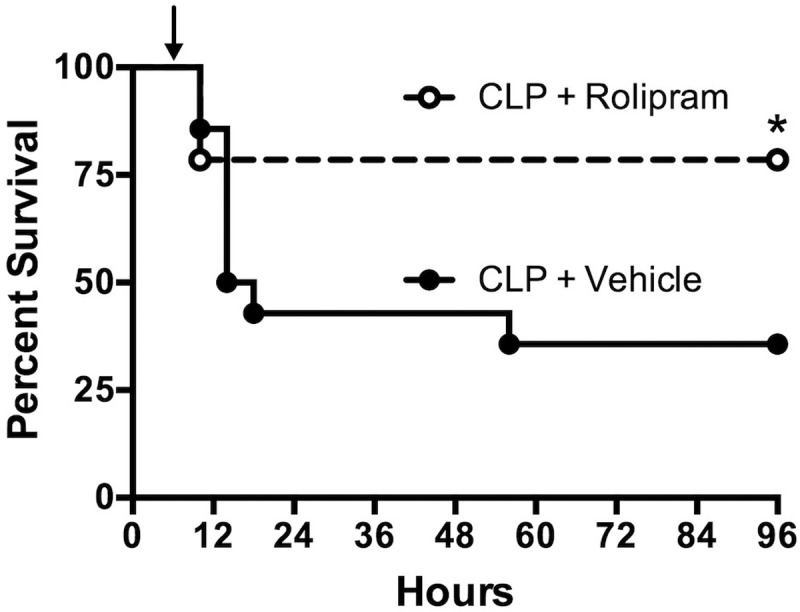
**Effect of delayed dosing of rolipram on survival.** Animals were subjected to CLP and then dosed with rolipram (0.1 mg/kg, ip) or vehicle at 6 h (indicated by the arrow). Survival was monitored through 96 h. Rolipram significantly improved 96-h survival compared to vehicle; ^∗^*p* < 0.05 by Log-rank test; *n* = 14 animal in each group.

The ability of rolipram, even with clinically relevant delayed dosing, to improve renal microvascular function and cardiac function suggested that rolipram might improve survival, the ultimate therapeutic endpoint. Rat pups subjected to CLP showed a 36% survival at 96 h (**Figure 10**). Treatment with a single dose of rolipram (0.1 mg/kg, ip) at 6 h post-CLP significantly improved survival to 78% with a log-rank hazard ratio of 0.317 (68% reduction in risk of death compared to vehicle).

## Discussion

There is growing appreciation that the adult animal is not the appropriate model for studying sepsis in the infant population (Solhaug et al., 2004; Seely et al., 2011; Wheeler et al., 2011; Deep et al., 2013; Fink and Warren, 2014; Efron et al., 2015). While no animal model can fully replicate the complexities of human sepsis, the CLP model in the rat pup displays key clinically relevant characteristics of human infant sepsis-induced MODS including increased lactate, microvascular hypoperfusion, cold shock with reduced cardiac output, and the rapid development of cardiac and renal dysfunction (type 5 CRS), all of which are associated with poor outcomes in this understudied patient population (Ceneviva et al., 1998; Aneja and Carcillo, 2011; Wheeler et al., 2011; De Backer et al., 2013; Casserly et al., 2015).

The clinical relevance of this model directed us to use it in preclinical studies to evaluate the therapeutic potential of the PDE4 inhibitor, rolipram. Our previous study in the adult aged mouse made septic by CLP showed that rolipram was effective in reversing renal injury (Holthoff et al., 2013). However, the adult mouse model is associated with profound hypotension, which is absent in the pup model (Seely et al., 2011). The differences in these rodent models are clinically relevant because approximately 60% of septic infants present with cold shock while approximately 90% of septic adults present with warm shock (hypotension and increased cardiac output) (Parker et al., 1987; Ceneviva et al., 1998). Hypotension in septic infants occurs later in the course of sepsis because infants compensate initially by vasoconstriction to maintain blood pressure (Ceneviva et al., 1998). This appears to also occur in septic pups, which receive fluids after surgery and again at 6 h to help maintain blood pressure. However, we have not measured blood pressure in septic pups beyond 18 h (Seely et al., 2011), a time when mortality is 50%. Inhibitors of PDE4 can reduce vascular resistance and protect the microcirculation (Carcillo et al., 1996; Holthoff et al., 2013), which is why preservation of renal peritubular capillary perfusion was used as the end-point for the dose-response study. An important finding was that the lowest most efficacious dose of rolipram in the septic adult mouse was lethal in the septic rat pup. Because inhibitors of PDE4 can decrease blood pressure (Thomas et al., 2001), higher doses could hinder cardiovascular compensation in septic pups and increase mortality. While additional studies are required to understand whether high-dose toxicity was due to hypotension, species differences, or pharmacodynamic/pharmacokinetic differences (Krause and Kuhne, 1988), the results of our dose-response study emphasized the importance of evaluating potential therapies in the appropriate age model.

Kidney injury developed rapidly following CLP. The decrease in renal peritubular capillary perfusion and GFR were profound as early as 6 h, a time when lactate levels were increased as well as the inflammatory cytokines TNF-α and IL-1β (Seely et al., 2011). Since therapy for the septic infant is begun only after the onset of symptoms, we evaluated rolipram using a delayed dosing paradigm begun at 6 h post-CLP. Rolipram not only restored renal capillary perfusion at 18 h, it restored renal perfusion velocity and prevented microvascular leakage. However, rolipram did not prevent mitochondrial oxidant generation by the tubular epithelium and did not restore GFR. This finding suggests that improving renal microvascular perfusion alone is not enough to restore GFR when the tubular epithelium is under oxidative stress.

We observed that impairment of cardiac function, as measured by cardiac catheterization, developed rapidly following CLP. Cardiac catheterization is the gold standard to assess systolic and diastolic cardiac function in pediatric patients. At 6 h LV dP/dt max, an indicator of systolic function was slightly but significantly decreased. This was not observed by echocardiography, which showed no significant changes in shortening fraction or ejection fraction at 6 h. Several studies demonstrate limitations of standard echocardiographic measures like M-Mode fractional shortening and ejection fraction in assessment of LV systolic dysfunction in septic children. Ranjit et al. (2014) showed that the majority of pediatric patients (68%) with septic shock had only mild to moderate impairment of LV function measured by echocardiogram. These findings are also consistent with several adult studies (Poelaert et al., 1997; Dalla et al., 2015). Although pediatric patients with septic shock often have compromised LV function, non-invasive assessment of ventricular dysfunction by echocardiogram might fail to detect the early changes before ejection fraction and cardiac output decrease. The decrease in stroke volume and cardiac output observed by echocardiography at 6 h in septic pups could be also related to diastolic dysfunction and limited filling related to low ventricular compliance. At 18 h, echocardiography showed slight but significant decreases in fractional shortening and ejection fraction, and a sustained decrease in stroke volume. These data suggest a worsening of systolic cardiac function over time.

Impaired LV diastolic function is a frequent occurrence in septic infants (Raj et al., 2014; Sankar et al., 2014). In agreement with these clinical findings, pups showed significant decreases in LV dP/dt min and increases in Tau at 6 h following CLP, suggesting severe diastolic dysfunction due to a direct effect of sepsis on cardiac muscle. Several studies have shown that Tau is a preload-independent measure of isovolumic relaxation of LV. It is independent of end-diastolic pressure, peak LV pressure, stroke volume and heart rate. Although Tau might be slightly influenced by very high or very low heart rate (Raff and Glantz, 1981), we did not observe significant changes in heart rate among groups during the catheterization measurements.

The decrease in LV function was also associated with elevated cTnl, a biomarker for cardiac injury. This finding is consistent with what has been reported in pediatric septic patients. Fenton et al. (2004) showed that elevated serum levels of troponin are associated with cardiac dysfunction in pediatric patients with septic shock and correlates with the severity of illness. Moreover, levels of cTnI are highest early in the course of sepsis (Fenton et al., 2004). Interestingly, LV function is often reversible in septic children and did recover by 18 h in septic pups. However, the nature of these experiments required that different groups of animals be used for measurements of cardiac function at 6 and 18 h. Moreover, measurements at 18 h were made in survivors, which were only approximately 50% of septic pups at that time point. Consequently, the data cannot suggest a true rate of recovery.

The ability of rolipram to prevent LV dysfunction at 6 h was impressive. To determine whether rolipram might be clinically useful in improving cardiac contractility in septic infants as are PDE3 inhibitors (Barton et al., 1996; Irazuzta et al., 2007; Meyer et al., 2011), rolipram was administered at the time of CLP and cardiac catheterization was performed at 6 h. All the measurements of systolic and diastolic function by cardiac catheterization were improved with no change in the mean arterial pressure. These findings were confirmed by echocardiography using clinically relevant delayed administration of rolipram. For these experiments, rolipram was given at 6 h and echocardiography was performed at 18 h. Even with delayed therapy, rolipram reversed the decline in stroke volume and cardiac output and prevented the decreases in fractional shortening and ejection fraction. These improvements in cardiac function suggest direct effects of rolipram on cardiomyocyte contractility and relaxation; however, additional studies are required to confirm this.

There are relatively few detailed studies describing the development of CRS in infants with sepsis (Jefferies and Goldstein, 2013) and, to our knowledge, none in a relevant animal model of infant sepsis. The rapid development of cardiac and renal dysfunction in this rat pup model is consistent with type 5 CRS. In both animal models of sepsis and postmortem septic human adult tissue samples, the generally mild changes in kidney and heart morphology observed by light microscopy do not explain the severity of kidney or heart dysfunction (Smeding et al., 2012; Takasu et al., 2013). In other forms of CRS, crosstalk between the heart and kidneys, which can worsen organ injury are better understood (Doi and Rabb, 2016). During sepsis, heart and kidney dysfunction occur essentially simultaneously so it is difficult to experimentally uncover interdependence. For example, the ability of rolipram to improve renal microvascular perfusion may have been, at least partially, due to an improvement in cardiac output. Studies from other types of CRS suggest that the sustained decline in cardiac and kidney function may be influenced by heart–kidney crosstalk through tissue release of cytokines and vasoactive hormones (Doi and Rabb, 2016). Additional studies are required to address this directly in this model.

The ability of rolipram to improve cardiac function and the renal microcirculation, even without improving GFR, is an important finding because it has been shown that organ failures have a cumulative effect on mortality and that reducing even one organ dysfunction can improve the prognosis of the septic patient (Martin et al., 2003). Still, the ultimate test of a new therapeutic for the septic patient is its effect on mortality. A single dose of rolipram administered after the onset of symptoms of CRS significantly improved 4-days survival. While additional studies are required to establish whether additional doses could have improved renal function and survival even further, the therapeutic potential of rolipram in this model of infant sepsis is encouraging.

There are several important limitations of our studies. One limitation is fluid status. All pups received fluids at the end of surgery (25 ml/kg of saline) and again at 6 h (40 ml/kg of saline). Both systolic and diastolic dysfunctions are dependent on load and fluid status (Vasan and Levy, 2000). Intravascular volume depletion reduces LV filling and could therefore result in what appears to be diastolic dysfunction. We could confirm that mean arterial pressure was unchanged in septic pups as we reported previously (Seely et al., 2011), and that hematocrit was unchanged; however, actual intravascular volume status was unknown. Regardless, rolipram clearly improved LV function and ultimately cardiac output. A second limitation is the use of short axis M-mode echocardiography to assess cardiac output in rat pups. While short axis M-mode measurements have been used previously in mice and children with some correlation with invasive measurements of cardiac output (Walther et al., 1986; Wallerson et al., 1990; Tournoux et al., 2011), these data must be interpreted cautiously. A third limitation is that we did not specifically address the mechanism of action of rolipram. PDE4 isoforms are present in rat and human cardiomyocytes (Johnson et al., 2012; Soler et al., 2015); however, the potential beneficial effect of PDE4 inhibitors in the human heart during sepsis remains to be determined (Eschenhagen, 2013). PDE4 is also expressed in various cell types in the kidney (Cheng and Grande, 2007). We know rolipram reached in the kidney even with delayed administration despite reduced GFR because renal capillary leakage was prevented. However, we do not know if rolipram reached pharmacologically active concentrations in the heart or whether the levels of cyclic nucleotides were increased. These studies are currently underway. While we cannot rule out the possibility that the anti-inflammatory actions of rolipram contributed to the clinically relevant improvements in organ function and survival, they cannot account for all its effects because inflammatory cytokines were already elevated at the time rolipram was administered (Seely et al., 2011).

In summary, our studies are the first to characterize cardiac and renal dysfunction in an animal model of infant sepsis-induced CRS. Our findings with rolipram suggest that PDE4 inhibitors, by targeting microvascular leakage and the inflammatory process in addition to cardiac contractility and vascular tone (Sanz et al., 2007; Schick et al., 2012), may offer therapeutic advantages over PDE3 inhibitors, such as milrinone, which is currently recommended for pediatric “cold shock” in the current American College of Critical Care Medicine Guidelines for Septic Shock Resuscitation (Barton et al., 1996; Irazuzta et al., 2007; Brierley et al., 2009; Meyer et al., 2011). These additional therapeutic targets would have added benefits in infants with sepsis.

## Conclusion

While early goal-directed therapy intended to maintain systemic hemodynamics with the intent of preserving organ perfusion has been shown to reduce mortality in septic infants (Brophy, 2008; Brierley et al., 2009), the lack of targeted therapies remains a limitation of current management of the septic infant. Clinical trials for sepsis therapy have often resulted in a worsening of patient outcomes because preclinical studies often do not use age-appropriate models nor evaluate therapy for multiple organ dysfunction (Fink and Warren, 2014; Cohen et al., 2015; Sims et al., 2016). Using our model of type 5 CRS in the rat pup, we present preclinical pharmacological evidence that the PDE4 inhibitor rolipram may offer a more targeted approach to treating the septic infant.

## Author Contributions

CS, SM, SS, TN, and PM were involved in the study design and conception of the manuscript. CS, SM, and SS conducted the animal experiments and were responsible for data collection and analysis along with DZ, TN, NG, and PM. All authors contributed to the writing of the manuscript and approved the final manuscript.

## Conflict of Interest Statement

The authors declare that the research was conducted in the absence of any commercial or financial relationships that could be construed as a potential conflict of interest. The reviewer YZ and handling Editor declared their shared affiliation, and the handling Editor states that the process nevertheless met the standards of a fair and objective review.

## Abbreviations

BUN: blood urea nitrogen
cAMP: cyclic adenosine monophosphate
cGMP: cyclic guanosine monophosphate
CLP: cecal ligation and puncture
CRS: cardiorenal syndrome
cTnI: cardiac troponin I
DMSO: dimethyl sulfoxide
EBD: Evans Blue dye
FITC: fluorescein isothiocyanate
GFR: glomerular filtration rate
H&E: hematoxylin and eosin
IL: interleukin
ip: intraperitoneal
LV: left ventricle
PAS: periodic acid-Schiff
PDE: phosphodiesterase
RBC: red blood cell
sc: subcutaneous
TNF: tumor necrosis factor
